# Publisher Correction: Territoriality modulates the coevolution of cooperative breeding and female song in songbirds

**DOI:** 10.1038/s41559-026-03046-w

**Published:** 2026-03-18

**Authors:** Kate T. Snyder, Aleyna Loughran-Pierce, Nicole Creanza

**Affiliations:** 1 Department of Biological Sciences, Vanderbilt University, Nashville, TN USA; 2 Evolutionary Studies Initiative, Vanderbilt University, Nashville, TN USA; 3 School for Science and Math at Vanderbilt, Vanderbilt University, Nashville, TN USA

**Keywords:** Social evolution, Sexual selection

Publisher Correction to: *Nature Ecology & Evolution* 10.1038/s41559-026-02981-y, published online 25 February 2026.

Since the version of the article initially published, several instances of “0” have been removed from the heatmap in Fig. 4d to make it consistent with the figure legend. The original figure is available for comparison in the Supplementary information accompanying this amendment. This correction has been made to the HTML and PDF versions of the article.

## Supplementary information

Original, uncorrected Fig. 4

