# Supplementary material for: Territoriality modulates the coevolution of cooperative breeding and female song in songbirds
Source: Nat Ecol Evol. 2026 Feb 25;10(3):536–49. doi: 10.1038/s41559-026-02981-y (PMC12971482; doi:10.1038/s41559-026-02981-y)
Supplement: Supplementary file 1 — Supplementary Tables 1–25, Figs. 1 and 2, Methods Text and Results Text. [file 41559_2026_2981_MOESM1_ESM.pdf]

nature ecology & evolution

Supplementary information https://doi.org/10.1038/s41559-026-02981-y

Territoriality modulates the coevolution of cooperative breeding and female song in songbirds

In the format provided by the authors and unedited

Table of Contents

Supplementary Table 1: Binary states of each sociality metric with species counts.
Supplementary Table 2: Species counts for each combination of female song presence and breeding system and sociality metrics
Supplementary Table 3: Results of phylANOVA testing whether song features differ between states of different sociality traits.
Supplementary Table 4: Results assessing whether rates of song-feature evolution differ based on the predicted ancestral state of multiple sociality metrics.
Supplementary Table 5: Results assessing whether the rate of song repertoire size evolution differs based on the predicted ancestral state of cooperative breeding status across subsets of species, iteratively removing each family with more than 2 species in the analysis.
Supplementary Table 6: Results assessing whether the rate of song repertoire size evolution differs based on the predicted ancestral state of several multi-state traits
Supplementary Table 7: Overlap of stochastic character map simulations of cooperative breeding and female song using a jackknife analysis in which each family was removed from the datasets in turn.
Supplementary Table 8: Results of overlapping simulations of ancestral character history for cooperative breeding and female song using alternative phylogenies, alternative methods of determining cooperative breeding, and cooperative breeding classifications based on individual sources
Supplementary Table 9: Species counts for each combination of the social system classification from the current study and individual sources used to generate the database, separated by female song presence or absence based on the classification in the current study.
Supplementary Table 10: Results of overlapping simulated ancestral character histories for binary social variables with those of cooperative breeding.
Supplementary Table 11: Overlap of stochastic character map simulations of female song and four-state social traits.
Supplementary Table 12: Distribution of species across cooperative breeding and female song states within different territorial contexts.
Supplementary Table 13: Tests for sampling biases in female song and cooperative breeding data availability.
Supplementary Table 14: Stratified downsampling calculations to correct for sampling biases in the comparative dataset.
Supplementary Table 15: Results of phylogenetic generalized linear models using alternative cooperative breeding classifications with female song as the response variable.
Supplementary Table 16: Results of of phylogenetic generalized linear models using alternative cooperative breeding classifications with cooperative breeding as the response variable.
Supplementary Table 17: Best-fit models from a phylogenetic generalized linear modeling analysis that included female song, cooperative breeding, year-round territoriality, and body mass.
Supplementary Table 18: Phylogenetic generalized linear model comparisons of cooperative breeding versus alternative social predictors of female song.
Supplementary Table 19: Rates of transition between states of binary traits obtained using Ancestral Character Estimation (R Package ‘ape’, function ‘ace’) with the Equal Rates (ER) and All Rates Different (ARD) models, calculated using Oscine species only.
Supplementary Table 20: Comparing values of continuous and ordinal variables, scaled and centered across female song and cooperative breeding groups using phylANOVA.
Supplementary Table 21: Best phylogenetic generalized linear model predicting female song after stepwise predictor addition, using territory strength as the territorial categorization.
Supplementary Table 22: Best phylogenetic generalized linear model predicting cooperative breeding after stepwise predictor addition, using territory strength as the territorial categorization.

Supplementary Table 23: Best phylogenetic generalized linear model predicting female song after stepwise predictor addition, using year-round territoriality as the territorial categorization.
Supplementary Table 24: Best phylogenetic generalized linear model predicting cooperative breeding after stepwise predictor addition, using year-round territoriality as the territorial categorization.
Supplementary Table 25: Species counts with each combination of trait states for traits geographic region, cooperative breeding, and female song.

Supplementary Figure 1: Directed acyclic graph plots of all model structures tested in all phylopath analyses.
Supplementary Figure 2: Transition plots comparing evolution of female song with other sociality metrics.

Supplementary Methods Text

Supplementary Results Text

Supplementary Table 1: Binary states of each sociality metric with species counts. The last six rows provide the cooperative breeding assignments used in this manuscript. The number of species is the number of Oscine species with the noted classification, separated by state. In all analyses where the specific cooperative breeding classification method is not noted (i.e. in the main text), HighConfidence_Coop was used.

Original source column name	Source	Binarized column name in Supplementary Dataset 2	Source values assigned state "0" or "1"	State "0" and state "1" categories in this study	Number of oscine species assigned to state
colonial	Griesser et al (2023)	Griesser2023.Colonial01	acolonial (0)	Non-colonial	404
			colonial (1)	Colonial	83
parental.care.mode	Griesser et al (2017)	Griesser2017FamilialLiving	no_fam (0)	Non-familial living	577
			coop_families, family (1)	Familial living	626
grouping	Griesser et al (2023)	Griesser2023.Asocial0VsSocial1	asocial (0)	Asocial	47
			pair, small_groups, large_groups (1)	Social	409
grouping	Griesser et al (2023)	Griesser2023.GroupsLargerThanPair	asocial, pair (0)	Groups pair or smaller	88
			small_groups, large_groups (1)	Groups larger than pair	368
grouping	Griesser et al (2023)	Griesser2023.LargestGroupSizes	asocial, pair, small_groups (0)	Smaller groups	306
			large_groups (1)	Largest group sizes	150
social_bonds	Griesser et al (2023)	Griesser2023.LongSocialBonds	a-short, b-season (0)	Short social bonds	170
			c-long (1)	Season or shorter social bond	317
social_bonds	Griesser et al (2023)	Griesser2023.SeasonOrLongerSocialBonds	a-short (0)	Season or shorter social bonds	18
			b-season, c-long (1)	Long social bonds	469
caretakers	Griesser et al (2023)	Griesser2023.MoreThanTwoCaretakers	<= 2 (0)	Two or fewer caretakers	411
			> 2 (1)	More than two caretakers	76
caretakers	Griesser et al (2023)	Griesser2023.TwoOrMoreCaretakers	< 2 (0)	Fewer than two caretakers	24
			>= 2 (1)	Two or more caretakers	463
Final.polygyny	Snyder and Creanza (2019)	Final.polygyny	0 (Socially monogamous)	Social monogamy	626
			1 (Socially polygynous)	Social polygyny	108
Territory	Tobias et al (2016)	Territory_YearRound	1, 2 (0)	No territoriality, weak territoriality, or seasonal territoriality	3186
			3 (1)	Year-round territoriality	1246
Current study	Current study	TerritorialityWeakVsStrong	See methods	No territory or territory not actively defended	604
			See methods	Territory actively defended by individual, pair, or group	1560
Current study	Current study	HighConfidence_Coop	See methods	Non-cooperative	3904
			See methods	Cooperative	469
Current study	Current study	MeanCoopOmitTies	See methods	Non-cooperative	3940
			See methods	Cooperative	493
Current study	Current study	MeanCoopTie2Coop	See methods	Non-cooperative	3940
			See methods	Cooperative	554
Current study	Current study	MeanCoopTie2Noncoop	See methods	Non-cooperative	4001
			See methods	Cooperative	493
Current study	Current study	AnyCoopEqualsCoop	See methods	Non-cooperative	3859
			See methods	Cooperative	635
Current study	Current study	AnyNoncoopEqualsNoncoop	See methods	Non-cooperative	4060
			See methods	Cooperative	434

Supplementary Table 2: Species counts for each combination of female song presence and breeding system and sociality metrics. See Supplementary Table 1 for sources.

Sociality variables	Female Song Absent	Female Song Present	Non-cooperative Breeding	Cooperative Breeding
Non-colonial (breeds singly)	58	165	328	61
Colonial (breed in colonies)	12	35	74	9
Asocial (individuals usually on their own)	8	26	46	0
Social (individuals usually with at least one other individual)	56	165	325	70
Groups pair or smaller (usually on their own or in pairs)	15	49	85	0
Groups larger than pair (usually in groups of 3 or more)	49	142	286	70
Smaller groups (individuals usually in groups of 0-30 individuals)	34	139	230	65
Largest group sizes (individuals usually in groups of >30 individuals)	30	52	141	5
Short social bonds (bonds lasting less than one season)	11	2	18	0
Season or longer social bonds (bonds lasting one season or longer)	59	198	384	70
Season or shorter social bonds (bonds lasting at most one season)	34	61	161	3
Long social bonds (bonds lasting longer than one season)	36	139	241	67
Two or fewer caretakers (species exhibited mound-nesting (zero caretakers), uniparental care (one caretaker), or biparental care (two caretakers)	66	172	401	1
More than two caretakers (species had cooperative breeding with more than two caretakers)	4	28	1	69
Fewer than two caretakers (species exhibited mound-nesting (zero caretakers) or uniparental care (one caretaker))	12	6	24	0
Two or more caretakers (species had biparental care or cooperative breeding with more than two caretakers)	58	194	378	70
Non-familial living (parent–offspring associations do not extend beyond nutritional independence [includes non-kin cooperatively breeding species])	103	159	554	11
Familial living (offspring remain with their parents beyond nutritional independence [includes kin-based cooperatively breeding species])	59	147	345	249
Social monogamy (<5% of studied males exhibited polygyny)	102	166	531	72
Social polygyny (≥5% of males exhibited polygyny)	32	23	104	2
Asocial (usually on their own)	8	26	46	0
Pair (usually in pairs)	7	23	39	0
Small groups (usually 3 to 30 individuals (approximating personalized groups where group members know each other individually, including family groups)	19	90	145	65
Large groups (usually more than 30 individuals; more ephemeral or anonymous groups)	30	52	141	5
Weak, seasonal, or no territoriality	349	313	2807	218
Year-round territoriality	47	382	881	237
Weak or no territoriality	106	71	543	49
Active defense of territory (seasonally or year-round)	201	541	1162	258

Supplementary Table 3: Results of phylANOVA testing whether song features differ between states of different sociality traits. Continuous song traits were natural-log-transformed before analysis. Each trait pair (a combination of a discrete social trait and a continuous song trait) was tested using phytools::phylANOVA() for 50,000 simulations each. All song traits were tested relative to our main cooperative breeding dataset (HighConfidence_Coop, denoted in pink), but only song repertoire size was tested with other classification methods (yellow), with other social traits (blue), or with data obtained from individual cooperative breeding sources (purple) since none of the other song traits (syllable repertoire size, syllables per song, song duration, inter-song interval) showed clear trends relative to cooperative breeding in either phylANOVA or Brownie.

Social Trait	# of states of social trait	Song Trait	Number of species with data for both the social and song traits	phylANOVA p-value
HighConfidence_Coop	2	Syllable.rep.final	118	0.34825
HighConfidence_Coop	2	Song.rep.final	209	0.9772
HighConfidence_Coop	2	Syll.song.final	163	0.82805
HighConfidence_Coop	2	Duration.final	218	0.35155
HighConfidence_Coop	2	Interval.final	123	0.9097
MeanCoopTie2Noncoop	2	Song.rep.final	217	0.96575
MeanCoopTie2Coop	2	Song.rep.final	217	0.45575
MeanCoopOmitTies	2	Song.rep.final	214	0.9358
AnyCoopEqualsCoop	2	Song.rep.final	217	0.7963
AnyNoncoopEqualsNoncoop	2	Song.rep.final	217	0.77096
social_system_Griesser2017	3	Song.rep.final	156	0.93704
social_system_incl_nk_coop_Griesser2017	4	Song.rep.final	156	0.66026
grouping	4	Song.rep.final	127	0.72988
Griesser2023.Colonial01	2	Song.rep.final	129	0.79964
Griesser2023.MoreThanTwoCaretakers	2	Song.rep.final	129	0.85682
Griesser2023.LongSocialBonds	2	Song.rep.final	129	0.65492
Griesser2023.GroupsLargerThanPair	2	Song.rep.final	127	0.3345
Griesser2023.TwoOrMoreCaretakers	2	Song.rep.final	129	0.67844
Griesser2023.Asocial0vsSocial1	2	Song.rep.final	127	0.26796
Griesser2023.LargestGroupSizes	2	Song.rep.final	127	0.56416
Griesser2023.SeasonOrLongerSocialBonds	2	Song.rep.final	129	0.64316
Griesser2017FamilialLiving	2	Song.rep.final	156	0.78656
Territory (Tobias et al 2016)	3	Song.rep.final	216	0.58884
Territory_YearRound	2	Song.rep.final	216	0.4243
TerritorialityWeakVsStrong	2	Song.rep.final	159	0.53198
Cooperative breeding classifications from Biagolini et al (2017)	2	Song.rep.final	73	0.8115
Cooperative breeding classifications from Downing et al (2015)	2	Song.rep.final	71	0.3987
Cooperative breeding classifications from Cockburn (2016)	2	Song.rep.final	181	0.43534
Cooperative breeding classifications from Griesser et al (2017)	2	Song.rep.final	156	0.53256
Cooperative breeding classifications from Dale (2015)	2	Song.rep.final	199	0.61664
Cooperative breeding classifications from Cornwallis (2017)	2	Song.rep.final	204	0.6146
Cooperative breeding classifications from Jetz & Rubenstein (2011)	2	Song.rep.final	215	0.2319

Supplementary Table 4: Results assessing whether rates of song-feature evolution differ based on the predicted ancestral state of multiple binary sociality metrics (for multi-state metrics, see Supplementary Table 6). For each combination of song feature and sociality metric, out of 500 simulations per combination run using the Brownie algorithm (brownie.lite, R package: phytools), the Higher Rate State is determined as the discrete state in which the continuous trait evolved faster in at least 50% of the simulations, and the Lower Rate State is the other state. Cells highlighted in green indicate that at least 95% of simulations followed that Higher/Lower rate trend. *p*-values are derived from likelihood ratio tests (one-sided, df=1) based on the median log-likelihoods for the more complex and more simple models for each analysis. (*p*-values <0.05 highlighted in yellow). No adjustments were made for multiple comparisons. Results are provided for cooperative breeding system for all song features (pink). Song repertoire, syllable repertoire, and syllables per song each have at least one metric that is sufficiently significant, so we repeated the analysis using the minimum and maximum values per species for each; only song repertoire size had consistently significant results using min/max values, as well as across 500 iterations of resampled min/median/max values (orange), so we can only confidently conclude that song repertoire size appears to evolve at a significantly higher rate in non-cooperative breeding lineages than in cooperatively breeding lineages. We then also tested for state-dependent rates of song repertoire size evolution using alternative methods of cooperative breeding classification (yellow), our alternative consensus phylogeny (light green), multiple unique tree topographies (200 unique trees with the Hackett backbone from BirdTree.org, 20 simulations per tree; light green), other measures of sociality from Griesser et al (2017) and Griesser et al (2023) (see Supplementary Table 1; blue), and cooperative breeding data from individual sources (purple). The names of the social traits and song traits correspond to column names in Supplementary Datasets 1 and 2. Species counts are in Supplementary Table 3.

Binary Social Trait	Continuous Song Trait	Higher Rate State	Lower Rate State	Fraction of Sims Following Trend	Brownie *p*-value
HighConfidence_Coop	Syllable.rep.final	Non-Cooperative	Cooperative	0.982	0.136
HighConfidence_Coop	Song.rep.final	Non-Cooperative	Cooperative	0.994	0.024
HighConfidence_Coop	Syll.song.final	Cooperative	Non-Cooperative	0.906	0
HighConfidence_Coop	Duration.final	Cooperative	Non-Cooperative	0.704	0.0887
HighConfidence_Coop	Interval.final	Non-Cooperative	Cooperative	0.884	0.3797
HighConfidence_Coop	Syllable.rep.min	Non-Cooperative	Cooperative	0.988	0.1447
HighConfidence_Coop	Syllable.rep.max	Non-Cooperative	Cooperative	0.808	0.2336
HighConfidence_Coop	Song.rep.min	Non-Cooperative	Cooperative	0.992	0.026
HighConfidence_Coop	Song.rep.max	Non-Cooperative	Cooperative	0.994	0.028
HighConfidence_Coop	Syll.song.min	Cooperative	Non-Cooperative	0.696	0.2536
HighConfidence_Coop	Syll.song.max	Cooperative	Non-Cooperative	0.934	0
HighConfidence_Coop	Song repertoire (resampled min/median/max values)	Non-Cooperative	Cooperative	0.993	0.0332
MeanCoopTie2Noncoop	Song.rep.final	Non-Cooperative	Cooperative	0.992	0.0062
MeanCoopTie2Coop	Song.rep.final	Non-Cooperative	Cooperative	1	0.0029
MeanCoopOmitTies	Song.rep.final	Non-Cooperative	Cooperative	0.994	0.0077
AnyCoopEqualsCoop	Song.rep.final	Non-Cooperative	Cooperative	0.89	0.2222
AnyNoncoopEqualsNoncoop	Song.rep.final	Non-Cooperative	Cooperative	0.984	0.0194
HighConfidence_Coop (pooled results across 200 randomly sampled trees)	Song.rep.final	Non-Cooperative	Cooperative	0.98883333	0.032
HighConfidence_Coop (using alternative consensus tree)	Song.rep.final	Non-Cooperative	Cooperative	0.99	0.03
Griesser2023.Asocial0VsSocial1	Song.rep.final	Pair or group sociality	Asocial	1	0.0723
Griesser2023.GroupsLargerThanPair	Song.rep.final	Asocial or pair	Small or large groups	1	0.171
Griesser2023.LargestGroupSizes	Song.rep.final	Large groups	Asocial, pair, or small groups	0.53	0.6477
Griesser2023.SeasonOrLongerSocialBonds	Song.rep.final	Bonds last less than one season	Season or longer social bonds	0.89	0.3071

Griesser2023.LongSocialBonds	Song.rep.final	Bonds last one season or less	Multi-year bonds	1	0.0079
Griesser2017FamilialLiving	Song.rep.final	Non-Familial Living	Familial Living	0.998	0.0042
Griesser2023.Colonial01	Song.rep.final	Colonial	Non-Colonial	0.99	0.115
Griesser2023.MoreThanTwoCaretakers	Song.rep.final	Two or fewer caretakers	More than two caretakers	0.998	0.057
Territory_YearRound	Song.rep.final	Weak, seasonal, or no territoriality	Year-round territoriality	1	0.0106
TerritorialityWeakVsStrong	Song.rep.final	Weak or no territoriality	Strong territoriality	0.988	0.2165
Cooperative breeding classifications from Biagolini et al 2017 (transition rates based on source dataset)	Song.rep.final	Non-Cooperative	Cooperative	0.996	0.092
Cooperative breeding classifications from Biagolini et al 2017 (transition rates based on current study dataset)	Song.rep.final	Non-Cooperative	Cooperative	0.998	0.039
Cooperative breeding classifications from Downing et al (2015) (transition rates based on source dataset)	Song.rep.final	Non-Cooperative	Cooperative	0.994	0.198
Cooperative breeding classifications from Downing et al (2015) (transition rates based on current study dataset)	Song.rep.final	Non-Cooperative	Cooperative	0.988	0.156
Cooperative breeding classifications from Jetz and Rubenstein (2011) (transition rates based on source dataset)	Song.rep.final	Non-Cooperative	Cooperative	1	0.001
Cooperative breeding classifications from Jetz and Rubenstein (2011) (transition rates based on current study dataset)	Song.rep.final	Non-Cooperative	Cooperative	0.998	0.003
Cooperative breeding classifications from Cockburn (2016) (transition rates based on source dataset)	Song.rep.final	Non-Cooperative	Cooperative	0.996	0.003
Cooperative breeding classifications from Cockburn (2016) (transition rates based on current study dataset)	Song.rep.final	Non-Cooperative	Cooperative	0.994	0.003
Cooperative breeding classifications from Griesser et al (2017) (transition rates based on source dataset)	Song.rep.final	Non-Cooperative	Cooperative	1	0
Cooperative breeding classifications from Griesser et al (2017) (transition rates based on current study dataset)	Song.rep.final	Non-Cooperative	Cooperative	1	0
Cooperative breeding classifications from Dale (2015) (transition rates based on source dataset)	Song.rep.final	Non-Cooperative	Cooperative	0.952	0.137
Cooperative breeding classifications from Dale (2015) (transition rates based on current study dataset)	Song.rep.final	Non-Cooperative	Cooperative	0.91	0.178
Cooperative breeding classifications from Cornwallis (2017) (transition rates based on source dataset)	Song.rep.final	Non-Cooperative	Cooperative	0.996	0.021
Cooperative breeding classifications from Cornwallis (2017) (transition rates based on current study dataset)	Song.rep.final	Non-Cooperative	Cooperative	0.988	0.046

Supplementary Table 5: Results assessing whether the rate of song repertoire size evolution differs based on the predicted ancestral state of cooperative breeding status across subsets of species, iteratively removing each family with more than 2 species in the analysis. Each subset was run for 100 simulations using the Brownie algorithm (brownie.lite, R package: phytools). *p*-values are derived from likelihood ratio tests (one-sided, df=1) based on the median log-likelihoods for the more complex and more simple models for each analysis.

Song Trait	Higher rate of evolution in state:	Lower rate of evolution in state:	Fraction of simulations following trend	Brownie *p*-value	Number of Species In Subset Without Removed Family	Removed Family
Song.rep.final	Non-Cooperative	Cooperative	1	0.03	209	None
Song.rep.final	Non-Cooperative	Cooperative	1	0.025	202	Cardinalidae
Song.rep.final	Non-Cooperative	Cooperative	1	0.025	207	Certhiidae
Song.rep.final	Non-Cooperative	Cooperative	0.99	0.044	207	Corvidae
Song.rep.final	Non-Cooperative	Cooperative	1	0.033	162	Emberizidae
Song.rep.final	Non-Cooperative	Cooperative	1	0.03	206	Estrildidae
Song.rep.final	Non-Cooperative	Cooperative	1	0.031	203	Fringillidae
Song.rep.final	Non-Cooperative	Cooperative	1	0.018	206	Hirundinidae
Song.rep.final	Non-Cooperative	Cooperative	1	0.027	193	Icteridae
Song.rep.final	Non-Cooperative	Cooperative	0.98	0.035	205	Mimidae
Song.rep.final	Non-Cooperative	Cooperative	1	0.025	206	Motacillidae
Song.rep.final	Non-Cooperative	Cooperative	1	0.031	203	Muscicapidae
Song.rep.final	Non-Cooperative	Cooperative	0.99	0.033	197	Paridae
Song.rep.final	Non-Cooperative	Cooperative	0.99	0.02	182	Parulidae
Song.rep.final	Non-Cooperative	Cooperative	0.99	0.03	207	Reguliidae
Song.rep.final	Non-Cooperative	Cooperative	1	0.058	193	Sylviidae
Song.rep.final	Non-Cooperative	Cooperative	0.98	0.032	196	Troglodytidae
Song.rep.final	Non-Cooperative	Cooperative	1	0.033	195	Turdidae
Song.rep.final	Non-Cooperative	Cooperative	0.99	0.038	207	Viduidae
Song.rep.final	Non-Cooperative	Cooperative	1	0.028	198	Vireonidae

Supplementary Table 6: Results assessing whether the rate of song repertoire size evolution differs based on the predicted ancestral state of several multi-state traits: (A) group size, with four possible values, “asocial”, “pair”, “small groups”, and “large groups” (Griesser et al 2023), (B) territoriality, with three levels of classification, across 500 simulations using the Brownie algorithm (brownie.lite, R package: phytools), (C) combined territoriality strength and cooperative breeding across 1500 simulations, (D) social system, with three possible values (with the two non-familial groups merged, Griesser et al 2017), and (E) social system, with four possible values, “Cooperative, Familial”, “Cooperative, Non-familial”, “Non-cooperative, Familial”, and “Non-cooperative, Non-familial” (Griesser et al 2017). The columns “Higher Rate” and “Lower Rate” show which member of each pair of states was found to evolve at a higher and lower rate (respectively) the majority of the time, and the “Fraction of Simulations” columns indicate the frequency out of 500 simulations in which that pattern of higher- and lower-rate states was observed (those comparisons that were consistent in direction over at least 95% of simulations are highlighted in green). Overall, we found evidence that song repertoire size evolves at significantly different rates in lineages with different group sizes (Brownie likelihood-ratio test *p*-value 0.0004; n = 127 species), territoriality (Brownie likelihood-ratio test *p*-value = 0.012; n = 216 species), interactions between cooperative breeding and territory strength (Brownie likelihood-ratio test *p*-value = 0.011; n = 152 species), and both methods of categorizing social system (Brownie likelihood-ratio test 3-state *p*-value = 0.001, 4-state *p*-value = 0.002; both n = 156 species). See Extended Data Figure 2.

	Evolution of song repertoire size		
Social feature name	Higher Rate	Lower Rate	Fraction of Simulations
(A) grouping (Griesser et al 2023)	pair	asocial	1
	pair	large_groups	1
	pair	small_groups	1
	large_groups	asocial	0.998
	large_groups	small_groups	0.988
	asocial	small_groups	0.824
(B) Territory (Tobias et al, 2016)	Seasonal or weak territoriality (2)	Year-round territoriality (3)	1
	No territoriality (1)	Year-round territoriality (3)	1
	No territoriality (1)	Seasonal or weak territoriality (2)	0.996
(C) Cooperative Breeding x Weak vs Strong Territoriality (this paper)	Noncooperative_Weak	Cooperative_Strong	0.996
	Noncooperative_Strong	Cooperative_Strong	0.995
	Noncooperative_Weak	Noncooperative_Strong	0.987
	Cooperative_Weak	Cooperative_Strong	0.928
	Noncooperative_Weak	Cooperative_Weak	0.679
	Noncooperative_Strong	Cooperative_Weak	0.586
(D) “social system” three-state category from Griesser et al (2017)	Non-familial	Cooperative, Familial	0.998
	Non-familial	Non-cooperative, Familial	0.978
	Non-cooperative, Familial	Cooperative, Familial	0.64
(E) “social system including nonkin-coop” four-state category from Griesser et al (2017)	Non-cooperative, Non-familial	Cooperative, Familial	0.998
	Non-cooperative, Non-familial	Non-cooperative, Familial	0.988
	Non-cooperative, Non-familial	Cooperative, Non-familial	0.89
	Non-cooperative, Familial	Cooperative, Familial	0.694
	Cooperative, Non-familial	Cooperative, Familial	0.658
	Non-cooperative, Familial	Cooperative, Non-familial	0.558

Supplementary Table 7: Overlap of stochastic character map simulations of cooperative breeding and female song (data in HighConfidence_Coop and FemaleSong_Agg01 columns in Supplementary Datasets 2 and 3) using a jackknife analysis in which each family was removed from the datasets in turn. Each subset was run for 50 real simulations, 200 randomized-data simulations.

Removed family	Number of species in subset without removed family	Empirical *p*-value	Degree of association between traits			
			Non-cooperative breeding co-occurrence with female song absence	Non-cooperative breeding co-occurrence with female song presence	Cooperative breeding co-occurrence with female song absence	Cooperative breeding co-occurrence with female song presence
None	1041	<0.005	0.28	0.26	0.47	1
Acanthizidae	1020	0.005	0.325	0.255	0.37	1
Alaudidae	992	0.025	0.315	0.175	0.59	1
Campephagidae	1025	<0.005	0.32	0.23	0.295	1
Cardinalidae	1028	<0.005	0.235	0.185	0.51	1
Certhiidae	1038	0.015	0.22	0.315	0.51	1
Cinclosomatidae	1038	<0.005	0.255	0.3	0.565	1
Cisticolidae	974	<0.005	0.54	0.07	0.6	1
Climacteridae	1036	0.01	0.22	0.265	0.56	1
Colluricinclidae	1036	0.005	0.28	0.3	0.49	1
Corvidae	1027	0.015	0.29	0.325	0.54	1
Cracticidae	1033	0.005	0.245	0.26	0.635	1
Dasyornithidae	1038	0.02	0.225	0.275	0.415	1
Dicruridae	1029	<0.005	0.215	0.175	0.44	1
Emberizidae	979	0.02	0.275	0.22	0.585	1
Estrildidae	1017	<0.005	0.195	0.315	0.505	1
Eupetidae	1036	0.005	0.265	0.205	0.435	1
Fringillidae	1004	<0.005	0.315	0.205	0.22	1
Hirundinidae	1036	0.01	0.225	0.275	0.495	1
Icteridae	979	0.035	0.25	0.25	0.495	1
Laniidae	1031	0.01	0.24	0.22	0.455	1
Malaconotidae	1003	0.005	0.215	0.165	0.545	1
Maluridae	1027	0.01	0.225	0.315	0.385	0.995
Meliphagidae	1003	<0.005	0.23	0.235	0.49	1
Mimidae	1037	0.01	0.285	0.19	0.535	1
Monarchidae	1026	0.005	0.2	0.29	0.325	1
Motacillidae	1033	0.015	0.21	0.21	0.44	1
Muscicapidae	974	<0.005	0.21	0.305	0.52	1
Nectariniidae	1024	<0.005	0.12	0.275	0.445	1
Oriolidae	1033	0.005	0.29	0.175	0.475	1
Orthonychidae	1038	0.02	0.24	0.29	0.49	1
Pachycephalidae	1036	<0.005	0.25	0.3	0.395	1
Paradisaeidae	1016	0.005	0.335	0.17	0.605	1
Pardalotidae	1038	<0.005	0.28	0.205	0.42	1
Paridae	1034	0.015	0.225	0.275	0.54	1
Parulidae	1023	0.005	0.19	0.31	0.44	1
Passeridae	1031	<0.005	0.325	0.16	0.45	1
Petroicidae	1031	<0.005	0.345	0.2	0.48	1
Platysteiridae	1035	0.02	0.25	0.255	0.535	1
Ploceidae	1019	<0.005	0.285	0.24	0.315	1
Prunellidae	1038	0.005	0.19	0.275	0.435	1
Ptilonorhynchidae	1031	0.005	0.175	0.275	0.615	1
Pycnonotidae	1033	<0.005	0.255	0.19	0.415	1
Reguliidae	1038	0.01	0.17	0.25	0.455	1
Sittidae	1033	<0.005	0.37	0.115	0.4	1
Sturnidae	1021	<0.005	0.315	0.21	0.285	1
Sylviidae	1009	<0.005	0.235	0.23	0.495	1
Thraupidae	1011	0.005	0.245	0.215	0.585	1
Timaliidae	993	0.01	0.1	0.35	0.74	1

Supplementary Table 8: Results of overlapping simulations of ancestral character history for cooperative breeding and female song using alternative phylogenies, alternative methods of determining cooperative breeding, and cooperative breeding classifications based on individual sources, with transition rates determined based on either our full set of species or just the species present in that source, as noted. For each analysis, we ran 500 ancestral character mapping simulations with the real data and 500 with shuffled (randomized) data for comparison, except where otherwise noted. Empirical *p*-value indicates the *p*-value from our adapted Huelsenbeck et al (2003) method. Cells highlighted in yellow indicate significance at $p < 0.05$. Across the Trait A-Trait B stochastic character map pairs based on the real data, we found the median proportion of the phylogeny that each combination of traits occupied, then approximated the degree of association between trait states by calculating the fraction of randomized stochastic character map pairs that had a proportion of the tree occupying a given trait combination below the median of the real simulations. Cells highlighted in blue indicate that that trait combination was greater than the median of real simulations in <5% of randomized simulations (and thus the trait combination is less common than expected if traits were independent). Cells highlighted in red indicate that >95% of randomized simulations were less than the median of the real simulations (thus the trait combination is more common than expected if traits were independent). Colors in the first column follow Supplementary Table 4. For detailed breakdowns of the number of species in each state for each cooperative breeding classification method, see Supplementary Table 9.

			Degree of association between Trait A (female song absent versus female song present) and Trait B (non-cooperative versus cooperative breeding)			
Non-cooperative (0) vs. cooperative (1) data or phylogeny type	Number of species	Empirical *p*-value	Female song absent co-occurrence with non-cooperative breeding	Female song present co-occurrence with non-cooperative breeding	Female song absent co-occurrence with cooperative breeding	Female song present co-occurrence with cooperative breeding
Alternate consensus tree (mean.edge, if.absent = “ignore”)	1041	0.008	0.228	0.224	0.482	1
Multiple independent trees (200 trees, 20 simulations per tree)	1041	0.00075	0.462	0.131	0.514	1
Alternate cooperative breeding classification method: MeanCoopTie2Coop	1075	0.018	0.298	0.182	0.568	1
Alternate cooperative breeding classification method: MeanCoopOmitTies	1066	0.01	0.268	0.168	0.616	1
Alternate cooperative breeding classification method: AnyCoopEqualsCoop	1075	0.016	0.346	0.192	0.376	1
Alternate cooperative breeding classification method: AnyNoncoopEqualsNoncoop	1075	0.01	0.304	0.182	0.376	1
Alternate cooperative breeding classification method: MeanCoopTie2Noncoop	1075	0.012	0.254	0.136	0.734	1
Classifications from Cockburn 2006 (transition rates based on source dataset)	751	0.002	0.576	0.126	0.328	0.996
Classifications from Cockburn 2006 (transition rates based on current study dataset)	751	0.004	0.546	0.128	0.314	1
Classifications from Griesser et al 2017 (transition rates based on source dataset)	468	0.008	0.75	0.012	0.66	1

Classifications from Griesser et al 2017 (transition rates based on current study dataset)	468	0.004	0.602	0.004	0.912	1
Classifications from Dale 2015 (transition rates based on source dataset)	785	0.01	0.31	0.154	0.482	0.996
Classifications from Dale 2015 (transition rates based on current study dataset)	785	0.004	0.304	0.184	0.532	0.998
Classifications from Cornwallis 2017 (transition rates based on source dataset)	731	0.008	0.312	0.448	0.438	1
Classifications from Cornwallis 2017 (transition rates based on current study dataset)	731	0.01	0.316	0.486	0.314	0.998
Classifications from Jetz and Rubenstein 2011 (transition rates based on source dataset)	1058	0.04	0.222	0.092	0.848	1
Classifications from Jetz and Rubenstein 2011 (transition rates based on current study dataset)	1058	0.038	0.208	0.144	0.764	1
Classifications from Biagolini et al 2017 (transition rates based on source dataset)	114	0.16	0.512	0.228	0.53	0.974
Classifications from Biagolini et al 2017 (transition rates based on current study dataset)	114	0.238	0.48	0.196	0.648	0.936
Classifications from Downing et al 2015 (transition rates based on source dataset)	105	0.262	0.574	0.13	0.822	0.97
Classifications from Downing et al 2015 (transition rates based on current study dataset)	105	0.268	0.542	0.136	0.856	0.954

Supplementary Table 9: Species counts for each combination of the social system classification from the current study and individual sources used to generate the database, separated by female song presence or absence based on the classification in the current study. Color scheme is the same as Supplementary Table 4.

Column name	Social System State	Female Song Absent	Female Song Present
HighConfidence_Coop	Non-cooperative	358	558
	Cooperative	24	101
MeanCoopTie2Noncoop	Non-cooperative	366	572
	Cooperative	29	108
MeanCoopTie2Coop	Non-cooperative	361	568
	Cooperative	34	112
MeanCoopOmitTies	Non-cooperative	361	568
	Cooperative	29	108
AnyCoopEqualsCoop	Non-cooperative	353	538
	Cooperative	42	142
AnyNoncoopEqualsNoncoop	Non-cooperative	374	589
	Cooperative	21	91
Cooperative breeding classifications from Biagolini et al (2017)	Non-cooperative	26	71
	Cooperative	2	15
Cooperative breeding classifications from Downing et al (2015)	Non-cooperative	26	62
	Cooperative	2	15
Cooperative breeding classifications from Jetz and Rubenstein (2011)	Non-cooperative	101	191
	Cooperative	6	16
Cooperative breeding classifications from Cockburn (2016)	Non-cooperative	258	383
	Cooperative	24	86
Cooperative breeding classifications from Griesser et al (2017)	Non-cooperative	140	231
	Cooperative	22	75
Cooperative breeding classifications from Dale (2015)	Non-cooperative	268	382
	Cooperative	37	98
Cooperative breeding classifications from Cornwallis (2017)	Non-cooperative	279	411
	Cooperative	5	36
social_system_incl_nk_coop_Griesser2017	Cooperative/Familial	16	71
	Noncooperative/Familial	43	76
	Cooperative/Nonfamilial	6	4
	Noncooperative/Nonfamilial	97	155

Supplementary Table 10: Results of overlapping simulated ancestral character histories for binary social variables with those of cooperative breeding. For each pair of traits, we ran 500 ancestral character mapping simulations with the real data and 500 with shuffled data (i.e. with tip states in a randomized order) for comparison. We used methods adapted from [23] to estimate an empirical *p*-value (yellow indicates significance at $p < 0.05$). Degree of association between states: Across the pairs of stochastic character maps generated from the real social trait and cooperative breeding data, we found the median proportion of the phylogeny that each combination of traits occupied, then found the fraction of randomized stochastic character map pairs that had a proportion of the tree occupying a given trait combination below the median of the real simulations. Cells highlighted in blue indicate that, in <5% of the randomized-data simulations, the fraction of the tree occupying the indicated state combination was less than the median of simulations based on real data (and thus the state combination is less common than expected if traits were evolving independently). Cells highlighted in red indicate that, in >95% of randomized-data simulations, the state combination occupied a fraction of the tree less than the median of the real simulations (thus the trait combination is more common than expected if traits were evolving independently).

			Degree of association between states of Trait A (non-cooperative breeding versus cooperative breeding) and Trait B			
Social Trait (Trait B)	Number of species	Empirical p-value	Non-cooperative breeding co-occurrence with Social Trait = 0	Cooperative breeding co-occurrence with Social Trait = 0	Non-cooperative breeding co-occurrence with Social Trait = 1	Cooperative breeding co-occurrence with Social Trait = 1
Asocial (0) vs. social groups (1)	441	0.006	0.78	0.104	0.01	1
Asocial or pair (0) vs larger groups (1)	441	0.006	0.914	0.088	0.004	1
Asocial, pair, or small group (0) vs. large group (1)	441	<0.002	0.016	1	0.664	0.314
Social bonds lasting one season or less (0) vs longer than one breeding season (1)	472	<0.002	0.018	0.002	0.56	1
Non-familial (0) vs. familial living (1)	1159	<0.002	0.372	0.024	0	1
Non-colonial (0) vs. colonial living (1)	472	0.036	0.042	1	0.624	0.584
Weak or no territoriality (0) vs. strong territoriality (1)	2012	<0.002	0.41	0.686	0	1
Weak, seasonal, or no territoriality (0) vs. year-round territoriality (1)	4143	<0.002	0	1	1	1
Monogamy (0) vs. polygyny (1)	709	0.024	0.03	1	0.62	0.49
One or two caretakers (0) vs. more than two caretakers (1)	472	<0.002	0.094	0	0	1

Supplementary Table 11: Overlap of stochastic character map simulations of female song and four-state social traits. The degree of association between trait states was obtained by calculating the fraction of randomized stochastic character map pairs that had a proportion of the tree occupying a given trait combination below the median value from the real simulations. A) Combined classification of breeding system including kin living and cooperative breeding ("social system including nonkin-coop" from Griesser et al 2017). The empirical *p*-value comparing the overlap between estimated ancestral states of these traits over 1500 simulations is 0.0173, indicating that breeding system and female song co-occur significantly more than expected by chance. Cells highlighted in blue indicate that that trait combination was less than the median of real simulations in <5% of randomized simulations (and thus the trait combination is less common than expected if traits were independent). Cells highlighted in red indicate that >95% of randomized simulations were less than the median of the real simulations (thus the trait combination is more common than expected if traits were independent). Data correspond to Figure 3C. B) Group size classification ("grouping" from Griesser et al 2023). The empirical *p*-value over 1500 simulations is 0.267, indicating that female song is not significantly associated with group size. Sample sizes: (A) n = 468 species, (B) n = 255 species. Data correspond to Extended Data Figure 3B.

	Degree of association between traits	Female song state	
	Social system state	Absent	Present
A	Familial/Cooperative	0.607	1
	Familial/Non-cooperative	0.551	0.196
	Non-familial/Cooperative	0.342	0.028
	Non-familial/Non-cooperative	0.825	0.024
B	Asocial	0.622	0.464
	Pair	0.505	0.172
	Small groups	0.37	0.891
	Large groups	0.719	0.355

Supplementary Table 12: Distribution of species across cooperative breeding and female song states within different territorial contexts. The table shows raw species counts in our dataset for each combination of traits using two territorial classification schemes: (A) weak versus strong territoriality based on defensive behavior intensity, and (B) whether or not the species was territorial year-round. Cell shading indicates deviation from the expected counts under independence, calculated as (observed-expected)/expected, where blue indicates fewer species than expected and red indicates more species than expected if female song and cooperative breeding were distributed independently within each territorial category. These raw counts do not account for shared ancestral history, but the trends are consistent with the results of our phylogenetic comparative analyses. In each territory category, female song presence and cooperative breeding co-occurred more often than expected (ranging from 98% more than expected in the weak or no territoriality category to 0.9% more than expected in the year-round territoriality category). In both territory classification schemes, female song presence and cooperative breeding were more common in the more territorial category and co-occurred slightly more often than expected by chance, whereas in the less territorial category female song presence and cooperative breeding were less common overall but co-occurred much more often than expected by chance.

			Non-cooperative	Cooperative
A	Weak or no territoriality	Female Song Absent	101	3
		Female Song Present	56	10
	Strong territoriality	Female Song Absent	172	20
		Female Song Present	425	88
B	Weak, seasonal, or no territoriality	Female Song Absent	322	15
		Female Song Present	277	23
	Year-round territoriality	Female Song Absent	36	9
		Female Song Present	281	78

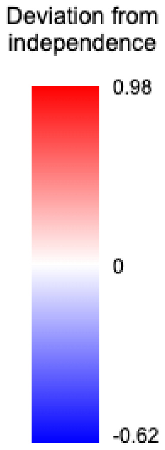

Supplementary Table 13: Tests for sampling biases in female song and cooperative breeding data availability. We tested whether data availability for female song presence/absence and cooperative breeding classification was biased with respect to various species traits that could confound evolutionary analyses. For each bias type, we tested the null hypothesis that data availability was independent of the trait in question. Chi-squared tests were used for categorical traits and Welch's t-tests for continuous traits. Significant biases ($p < 0.05$) indicate that species with certain trait values are over- or under-represented in our dataset. Geographic bias was tested using biogeographic regions from Jetz & Rubenstein (2011), with Holarctic (Nearctic + Palearctic) compared to tropical regions. Sexual dichromatism values were obtained from Dale et al. (2015) and sexual dimorphism from wing measurements in AVONET (Tobias et al. 2022). These detected biases were addressed through downsampling procedures (Supplementary Table 14) to ensure the robustness of our main findings.

Bias Type	Hypothesis	Test	Test statistic	df	p-value	Result
Cooperative breeding	Cooperative breeding species more likely to have female song data	Chi-squared	$\chi^2 = 2.18$	1	0.14	No bias in FS data
Coloniality	Colonial species more likely to be studied	Chi-squared<br>Chi-squared	$\chi^2 = 0.01$<br>$\chi^2 = 2.06$	1<br>1	0.91<br>0.15	No bias in FS data<br>No bias in CB data
Polygyny	Polygynous species more likely to be studied	Chi-squared<br>Chi-squared	$\chi^2 = 2.143$<br>$\chi^2 = 0.46$	1<br>1	0.143<br>0.498	No bias in FS data<br>No bias in CB data
Geographic	Holarctic species more likely to be studied	Chi-squared<br>Chi-squared	$\chi^2 = 56.80$<br>$\chi^2 = 0.74$	1<br>1	<0.001<br>0.785	**Bias detected in FS data**<br>No bias in CB data
Geographic x Cooperative breeding	Female song may be more or less likely to be studied based on the interaction between their geographic region and cooperative breeding status	Chi-squared	$\chi^2 = 56.92$	3	<0.001	**Bias detected in FS data** (Post-hoc tests found that non-cooperative holarctic species and cooperative tropical species are both overrepresented in the female song data)
Territoriality (Weak/Strong)	More territorial more likely to be studied	Chi-squared<br>Chi-squared	$\chi^2 = 58.67$<br>$\chi^2 = 31.49$	1<br>1	<0.001<br><0.001	**Bias detected in FS data**<br>**Bias detected in CB data**
Territory (Year-round)	Year-round territorial more likely to be studied	Chi-squared<br>Chi-squared	$\chi^2 = 89.22$<br>$\chi^2 = 39.18$	1<br>1	<0.001<br><0.001	**Bias detected in FS data**<br>**Bias detected in CB data**
Sexual dichromatism	Dichromatic species more likely to have FS data	t-test	$t = 3.46$	1789	<0.001	**Bias detected in FS data**
Sexual dimorphism	Dimorphic species more likely to have FS data	t-test	$t = -2.77$	1759	0.006	**Bias detected in FS data**

Supplementary Table 14: Stratified downsampling calculations to correct for sampling biases in the comparative dataset. For each identified bias (Supplementary Table 13), we calculated the number of species to remove to achieve balanced representation across groups. Current proportions show the fraction of species with complete female song and cooperative breeding data within each group. Target proportions represent the lower of the two groups being compared, ensuring conservative bias correction. For geographic biases, we performed two complementary corrections: removing overrepresented Holarctic non-cooperative species, and reducing the excess of tropical cooperative species among those with data. Although female song data availability was not significantly greater for cooperative breeding species globally, we performed an additional adjustment of the global cooperative breeding rate with female song data to match the population rate. All downsampling was performed independently 500 times.

Bias Category	Group to Downsample	Species to Remove	Current proportion of [Group to Downsample] with FS & CB data available	Target Proportion	Justification
Geographic	Holarctic non-cooperative	83	35.3% (209/592)	21.3%	Match tropical non-cooperative proportion
Geographic	Tropical cooperative	24	13.9% (112/804)	11.3%	Match overall tropical cooperative breeding rate
Cooperative breeding	Global cooperative	15	12.0% (125/1041)	10.7%	Match population cooperative breeding rate
Territoriality (Weak/Strong)	Strong territoriality	266	45.2% (705/1560)	28.1%	Match weak territoriality proportion
Territoriality (Year-round)	Year-round territoriality	155	32.4% (404/1246)	20.0%	Match seasonal/weak/no territory proportion
Sexual dichromatism	Continuous metric: preferentially downsample more dichromatic species	167	Remove species to approximately reduce the mean log(abs(plumage dimorphism)) (pre-normalization) of the subset for which we do have female song data (1.02) to the mean of the group for which we do not have data (0.86)		
Sexual dimorphism	Continuous metric: preferentially downsample more dimorphic species	71	Remove species to approximately reduce the mean % abs(wing dimorphism) (pre-normalization) of the subset for which we do have female song data (1.11) to the mean of the group for which we do not have data (1.04)		

Supplementary Table 15: Results of phylogenetic generalized linear models using alternative cooperative breeding classifications with female song as the response variable. The alternative cooperative breeding variables were inserted into the best-fit model from the tests performed using our primary cooperative breeding variable, Female Song ~ Cooperative Breeding * Territoriality (Weak vs Strong) + log(Mass) (centered and normalized). Cells display the mean Odds Ratio across 500 bootstrap iterations. Cells are filled if the empirical *p*-value is < 0.05, indicating that the bootstrapped odds ratio confidence interval does not include 1.0, with red cells indicating an odds ratio >1 and blue cells indicating an odds ratio <1. Significance is also indicated by symbols: *** p < 0.001, ** p < 0.01, * p < 0.05, ^ p < 0.1.

Cooperative Breeding Classifier	Response Variable	Species n	Intercept	Cooperative Breeding	Territoriality	Body Mass	CBxTerr
MeanCoopTie2Noncoop	Female Song	902	0.653^	4.370*	3.683***	1.411***	0.289*
MeanCoopTie2Coop	Female Song	902	0.654^	5.371***	3.567***	1.474*	0.222**
MeanCoopOmitTies	Female Song	895	0.631*	4.655*	3.642***	1.466***	0.272*
AnyCoopEqualsCoop	Female Song	902	0.534*	9.486***	4.477***	1.454***	0.110***
AnyNoncoop EqualsNoncoop	Female Song	902	0.663*	3.612*	3.637***	1.387*	0.379^
CockburnCoop	Female Song	650	0.597*	4.334**	4.952***	1.220^	0.290*
CockburnInferred	Female Song	864	0.648^	4.251***	3.868***	1.491**	0.256**
HighConf_Coop DefaultToCockburnInferred	Female Song	904	0.630*	4.941***	3.787***	1.490***	0.219**
Griesser2017Coop	Female Song	424	0.744	2.534	3.812***	1.776**	0.541
DaleCoop	Female Song	681	0.664^	6.738***	4.095***	1.198	0.136***
CornwallisCoop	Female Song	634	0.707	2.112^	3.978***	1.190	1.356
JetzCoopInclCockburn	Female Song	888	0.640*	4.706***	3.906***	1.477***	0.225*

Supplementary Table 16: Results of phylogenetic generalized linear models using alternative cooperative breeding classifications with cooperative breeding as the response variable. The alternative cooperative breeding variables were inserted into the best-fit model from the tests performed using our primary cooperative breeding variable, Cooperative Breeding ~ Female Song * Territoriality (Weak vs Strong). Cells display the mean Odds Ratio across 500 bootstrap iterations. Cells are filled if the empirical *p*-value is < 0.05, indicating that the bootstrapped odds ratio confidence interval does not include 1.0, with red cells indicating an odds ratio >1 and blue cells indicating an odds ratio <1. Significance is also indicated by symbols: *** $p < 0.001$, ** $p < 0.01$, * $p < 0.05$, ^ $p < 0.1$.

Response Variable	Species n	Intercept	Female Song	Territoriality	FSxTerr
MeanCoopTie2Noncoop	902	0.100**	5.514**	5.549**	0.181**
MeanCoopTie2Coop	902	0.099***	6.639**	7.077***	0.146**
MeanCoopOmitTies	895	0.104***	5.621***	5.844***	0.175***
AnyCoopEqualsCoop	902	0.134***	11.343***	9.033***	0.090***
AnyNoncoopEqualsNoncoop	902	0.115***	4.614***	4.454***	0.225***
CockburnCoop	650	0.099***	4.706***	4.230***	0.206***
CockburnInferred	864	0.179***	5.396***	5.656***	0.174***
HighConf_Coop DefaultToCockburnInferred	904	0.133***	6.244***	6.103***	0.152***
Griesser2017Coop	424	0.196**	2.707*	2.564*	0.339*
DaleCoop	681	0.113***	7.811***	5.390***	0.115***
CornwallisCoop	634	0.222^	1.493	0.994	0.757
JetzCoopInclCockburn	888	0.132**	5.663**	6.010**	0.165**

Supplementary Table 17: Best-fit models from a phylogenetic generalized linear modeling analysis that included female song, cooperative breeding, year-round territoriality, and body mass. Significant *p*-values ($p < 0.05$) are shown in bold and indicate that the 95% confidence interval of a predictor's odds ratio does not include 1.0.

Predictor (of Female Song)	*Odds Ratio*	*CI*	*p*	*Predictor (of Cooperative Breeding)*	*Odds Ratio*	*CI*	*p*
(Intercept)	0.77	0.51-1.11	0.180	(Intercept)	0.45	0.06-1.42	0.332
Cooperative Breeding	1.41	0.91-2.28	0.088	Female Song	1.44	1.15-1.93	**0.012**
Year-round territory	5.72	4.04-8.10	**<0.002**	Year-round territory	1.91	1.19-3.79	**<0.002**
log(Mass) (normalized	1.28	1.11-1.45	**0.004**	FemaleSong:Territory	0.59	0.30-1.05	0.116
Species	1041			Species	1041		
Model formula: Female Song ~ Cooperative Breeding + Territoriality (Year-round) + log(Mass)				Model formula: Cooperative Breeding ~ Female Song * Territory (Year-round)			

Supplementary Table 18: Phylogenetic generalized linear model comparisons of cooperative breeding versus alternative social predictors of female song. We compared cooperative breeding against each social variable using identical model structures with additive predictors (Female Song ~ [Social Variable] + Territoriality + Body Mass). Sample sizes represent species with complete data for both cooperative breeding and the social variable being compared. All ordinal or continuous social variables were standardized (scaled to have a standard deviation of 1 and mean of 0). The odds ratio for each predictor is reported as the mean and 95% CI across 500 bootstrap iterations. The *p*-value is determined empirically based on the fraction of bootstrap iterations where the predictor's odds ratio falls above or below 1.0.

			Results from model using **cooperative breeding** as the predictor (Female Song ~ Cooperative Breeding + Territoriality + Body Mass)				Results from model using the **social variable** as the predictor (Female Song ~ [Social Variable] + Territoriality + Body Mass)			
Territoriality variable	Social Variable	N Species	CB Model AIC	CB Odds Ratio	CB Odds Ratio 95% CI	CB *p*-value	Social Model AIC	Social Variable Odds Ratio	Social Variable Odds Ratio 95% CI	Social Variable *p*-value
Territoriality (Year-Round)	caretakers (normalized)	258	251.3	3.15	1.11-15.97	**0.044**	**242.9**	**9.13**	2.44-33.83	**0.02**
	Familial living	448	461.9	**1.50**	0.84-2.97	0.2	462.0	0.78	0.49-1.25	0.268
	Colonial living	258	251.3	**3.27**	1.12-18.82	**0.028**	252.5	1.22	0.62-2.17	0.472
	Grouping (normalized)	243	235.3	**3.31**	1.19-14.85	**0.016**	233.6	0.82	0.62-1.01	0.072
	Social bond duration (normalized)	258	251.3	**3.38**	1.20-18.00	**0.016**	251.2	1.15	0.84-1.57	0.264

Supplementary Table 19: Rates of transition between states of binary traits obtained using Ancestral Character Estimation (R Package ‘ape’, function ‘ace’) with the Equal Rates (ER) and All Rates Different (ARD) models, calculated using Oscine species only. The *p*-value was obtained using function anova() to compare the ER and ARD models. Bolded values indicate the rates used in simulations. Except where otherwise noted, all rates were calculated using the consensus tree calculated using default settings. See Supplementary Table 1 for “0” and “1” binary states for each trait.

trait	Rate of transition (Equal Rates; 0→1 = 1→0)	Rate of transition 0→1 (All Rates Different)	Rate of transition 1→0 (All Rates Different)	Log-Likelihood (Equal Rates model)	Log-Likelihood (All Rates Different model)	Likelihood ratio test *p*-value
Griesser2023.Colonial01	0.01269992	**0.01200588**	**0.08386504**	-213.22631	-186.39854	<0.001
Griesser2023.MoreThanTwoCaretakers	**0.00963502**	0.00966078	0.0162108	-164.98296	-164.52879	0.341
Griesser2023.LongSocialBonds	0.01945464	**0.04150603**	**0.00849845**	-241.33754	-230.45623	<0.001
Griesser2023.GroupsLargerThanPair	0.01094027	**0.04238356**	**0.00869378**	-168.51113	-155.24743	<0.001
Griesser2023.Asocial0VsSocial1	0.00778502	**0.06909473**	**0.00454807**	-144.41485	-112.31853	<0.001
Griesser2023.LargestGroupSizes	0.03826034	**0.03400503**	**0.0976376**	-293.0195	-272.68888	<0.001
Griesser2017FamilialLiving	0.05339407	**0.06052542**	**0.04661725**	-715.94102	-713.34626	0.023
Final.polygyny	0.01269635	**0.01199225**	**0.1090912**	-269.17593	-242.80781	<0.001
Territory_YearRound	0.02718114	**0.02175005**	**0.05177754**	-1806.8906	-1790.0845	<0.001
TerritorialityWeakVsStrong	0.01016844	**0.02487365**	**0.00718674**	-535.6235	-520.5321	<0.001
HighConfidence_Coop	0.01113287	**0.01071659**	**0.030594**	-1027.5535	-1019.6542	<0.001
FemaleSong_Agg01	0.03898356	**0.06718569**	**0.03828766**	-605.20332	-597.20892	<0.001
MeanCoopTie2Noncoop	0.01274633	**0.01190063**	**0.05181567**	-1164.7677	-1137.9685	<0.001
MeanCoopTie2Coop	0.01383931	**0.01343266**	**0.03829614**	-1218.3551	-1206.3045	<0.001
MeanCoopOmitTies	0.01222403	**0.01174713**	**0.03745765**	-1106.9228	-1092.8314	<0.001
AnyCoopEqualsCoop	0.01745169	**0.01751687**	**0.03761033**	-1420.5874	-1412.5426	<0.001
AnyNoncoopEqualsNoncoop	0.01143156	**0.01022155**	**0.06264396**	-1090.1805	-1048.4043	<0.001
HighConfidence_Coop (consensus tree with missing edges ignored in mean edge length calculation)	0.01084228	**0.01036728**	**0.03159119**	-1027.534	-1018.734	<0.001
FemaleSong_Agg01 (consensus tree with missing edges ignored in mean edge length calculation)	0.03697411	**0.06447600**	**0.03646347**	-603.8432	-595.8721	<0.001

Supplementary Table 20: Comparing values of standardized (i.e. scaled to have a standard deviation of 1 and center of 0) continuous and ordinal variables across female song and cooperative breeding groups using phylANOVA.

Variable	Evaluating differences between the states of trait:	Phyl-ANOVA p-value	Mean of group with trait state 0	SD of group with trait state 0	N Species with trait state 0	Mean of group with trait state 1	SD of group with trait state 1	N Species with trait state 1
abs(Latitude)	Female Song	0.58038	0.273	1.120	393	0.374	1.175	688
Number of caretakers	Female Song	**0.02552**	-0.326	0.340	70	**0.011**	0.903	200
grouping	Female Song	0.26032	**0.084**	1.141	64	-0.168	1.056	191
Body mass	Female Song	0.24686	0.067	1.052	397	0.251	0.965	697
Social bond duration	Female Song	**0.0016**	-0.460	1.331	70	**0.127**	0.872	200
Plumage dichromatism	Female Song	0.07304	0.272	0.999	397	-0.010	1.015	697
Wing dimorphism	Female Song	**0.04788**	**0.257**	1.191	375	-0.048	0.876	660
abs(Latitude)	Cooperative Breeding	0.86344	0.006	1.020	3847	-0.030	0.891	460
Number of caretakers	Cooperative Breeding	**2.00E-05**	-0.256	0.198	402	**1.471**	2.054	70
grouping	Cooperative Breeding	0.90294	-0.007	1.087	371	0.042	0.284	70
Body mass	Cooperative Breeding	**0.02648**	-0.040	0.982	3904	**0.363**	1.119	469
Social bond duration	Cooperative Breeding	**0.02124**	-0.106	1.041	402	**0.615**	0.365	70
Plumage dichromatism	Cooperative Breeding	0.06518	0.050	1.010	3848	-0.296	0.898	464
Wing dimorphism	Cooperative Breeding	0.36196	0.023	1.019	3547	-0.173	0.880	438

Supplementary Table 21: Best phylogenetic generalized linear model predicting female song after stepwise predictor addition, using territory strength as the territorial categorization. Predictors that were tested but not retained through the stepwise iterative process include latitude (distance from equator), geographic region, migration, and social mating system (polygyny). *p*-values less than 0.05 are in bold.

Predictors (of Female Song)	*Odds Ratio*	*CI*	*p*
(Intercept)	0.62	0.38-1.00	**0.048**
HighConfidence_Coop	5.87	4.01-8.87	**0.004**
TerritorialityWeakVsStrong	4.73	3.06-7.28	**<0.002**
logMass_normalized	1.31	1.05-1.63	**0.024**
PercentAbsLogWingDimorphism_normalized	0.83	0.71-0.97	**0.008**
logMaleFemalePlumageDiffAbs_normalized	0.85	0.71-1.00	0.056
HighConfidence_Coop:TerritorialityWeakVsStrong	0.22	0.16-0.36	**0.012**
Species	826		

Supplementary Table 22: Best phylogenetic generalized linear model predicting cooperative breeding after stepwise predictor addition, using territory strength as the territorial categorization. Predictors that were tested but not retained through the stepwise iterative process include latitude (distance from equator), geographic region, wing dimorphism, plumage dimorphism, migration, and social mating system (polygyny). *p*-values less than 0.05 are in bold.

Predictors (of Cooperative Breeding)	*Odds Ratio*	*CI*	*p*
(Intercept)	0.02	0.01-0.10	**<0.002**
FemaleSong_Agg01	11.74	3.42-27.39	**<0.002**
TerritorialityWeakVsStrong	2.41	0.85-5.83	0.080
Griesser2017FamilialLiving	14.61	5.28-35.33	**<0.002**
FemaleSong_Agg01:TerritorialityWeakVsStrong	0.07	0.02-0.29	**<0.002**
Species	407		

Supplementary Table 23: Best phylogenetic generalized linear model predicting female song after stepwise predictor addition, using year-round territoriality as the territorial categorization. Predictors that were tested but not retained through the stepwise iterative process include plumage dimorphism, migration, and social mating system (polygyny). *p*-values less than 0.05 are in bold.

Predictors (of Female Song)	*Odds Ratio*	*CI*	*p*
(Intercept)	0.73	0.45-1.12	0.148
HighConfidence_Coop	1.49	1.02-2.16	**0.040**
Territory_YearRound	7.02	4.94-10.45	**<0.002**
logMass_normalized	1.52	1.31-1.79	**<0.002**
abs_Latitude_normalized	1.23	1.05-1.42	**0.012**
PercentAbsLogWingDimorphism_normalized	0.87	0.75-0.98	**0.020**
GeographicRegion_JetzTropical	0.67	0.46-0.99	**0.044**
Species	961		

Supplementary Table 24: Best phylogenetic generalized linear model predicting cooperative breeding after stepwise predictor addition, using year-round territoriality as the territorial categorization. Predictors that were tested but not retained through the stepwise iterative process include latitude (distance from equator), geographic region, wing dimorphism, plumage dimorphism, migration, and social mating system (polygyny). *p*-values less than 0.05 are in bold.

Predictors (of Cooperative Breeding)	*Odds Ratio*	*CI*	*p*
(Intercept)	0.04	0.01-0.14	**<0.002**
FemaleSong_Agg01	1.93	1.00-4.12	0.052
Territory_YearRound	2.67	0.87-7.14	0.072
Griesser2017FamilialLiving	10.62	4.48-24.33	**<0.002**
FemaleSong_Agg01:Territory_YearRound	0.32	0.08-1.05	0.064
Species	448		

Supplementary Table 25: Species counts with each combination of trait states for traits geographic region (based on Jetz and Rubenstein 2011), cooperative breeding, and female song.

		Female Song	
Geographic Region	Cooperative Breeding	Absent	Present
Holarctic	non-cooperative	69	140
Tropical	non-cooperative	280	412
Holarctic	cooperative	3	8
Tropical	cooperative	20	92

Supplementary Figure 1: Directed acyclic graph plots of all model structures tested in all phylopath analyses.

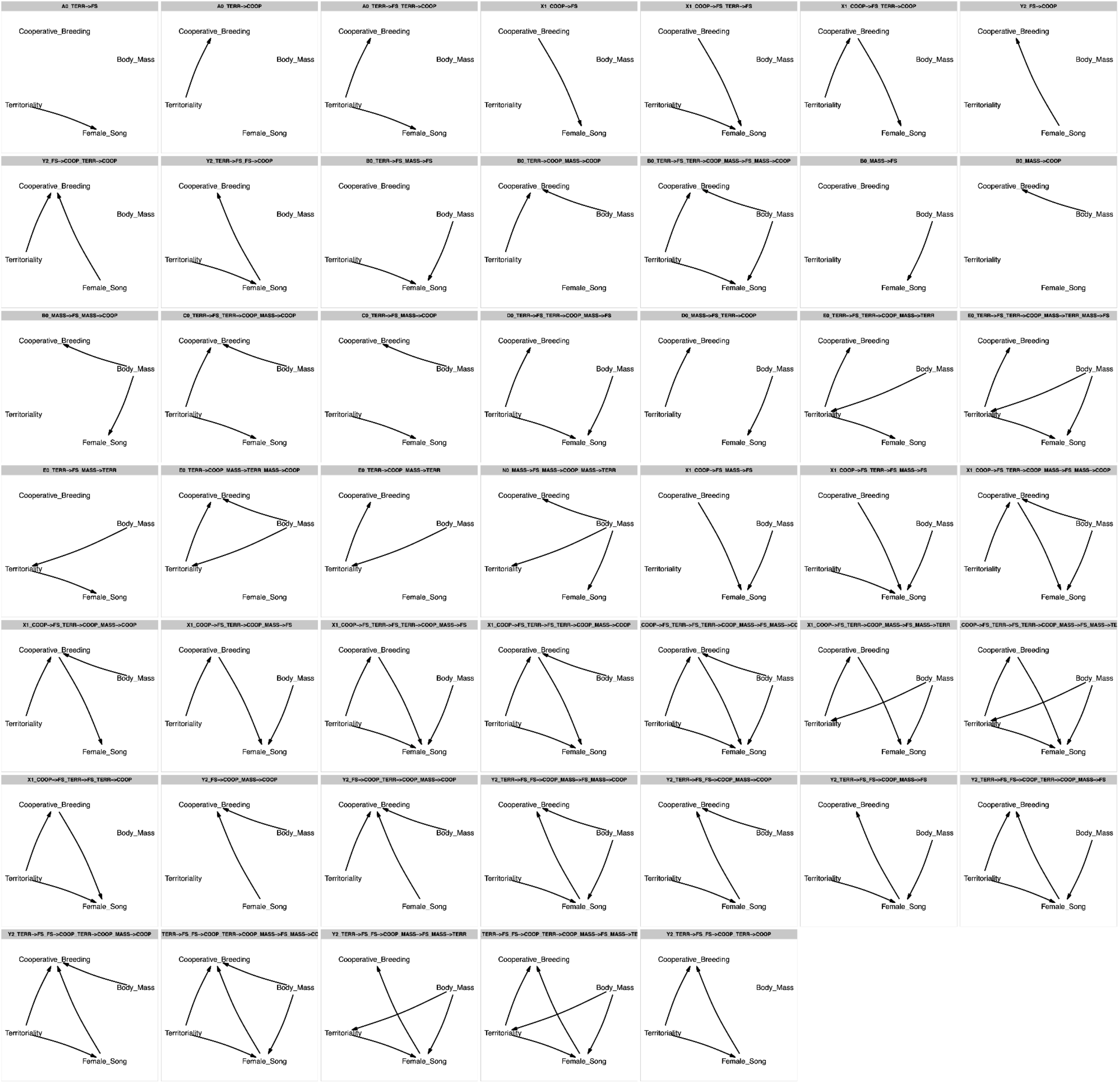

Supplementary Figure 2: Transition plots comparing evolution of female song with other sociality metrics: A) social group size larger than approximately 30 individuals, B) group size larger than pair, C) social bonds lasting longer than one year, D) social bonds lasting one season or longer, E) familial living, F) more than two caretakers, G) two or more caretakers, H) colonial living, and I) social mating system. Arrow weights vary as in Figures 4 and 5 and show the median log-scaled relative number of transitions that were estimated to occur across 500 simulations. Arrow color denotes the median difference from the expected number of transitions across simulations, with red indicating that the observed number of state switches was greater than expected, and blue indicating fewer state switches than expected. Asterisks indicate that the percent of simulations where the number of transitions is greater (red arrows) or less (blue arrows) than the calculated expected number of transitions for the simulation in question in >95% of simulations (**) or 90-95% of simulations (*). Sample sizes: A) n = 255 species, B) n = 255 species, C) n = 270 species, D) n = 270 species, E) n = 468 species, F) n = 270 species, G) n = 270 species, H) n = 270 species, I) n = 323 species.

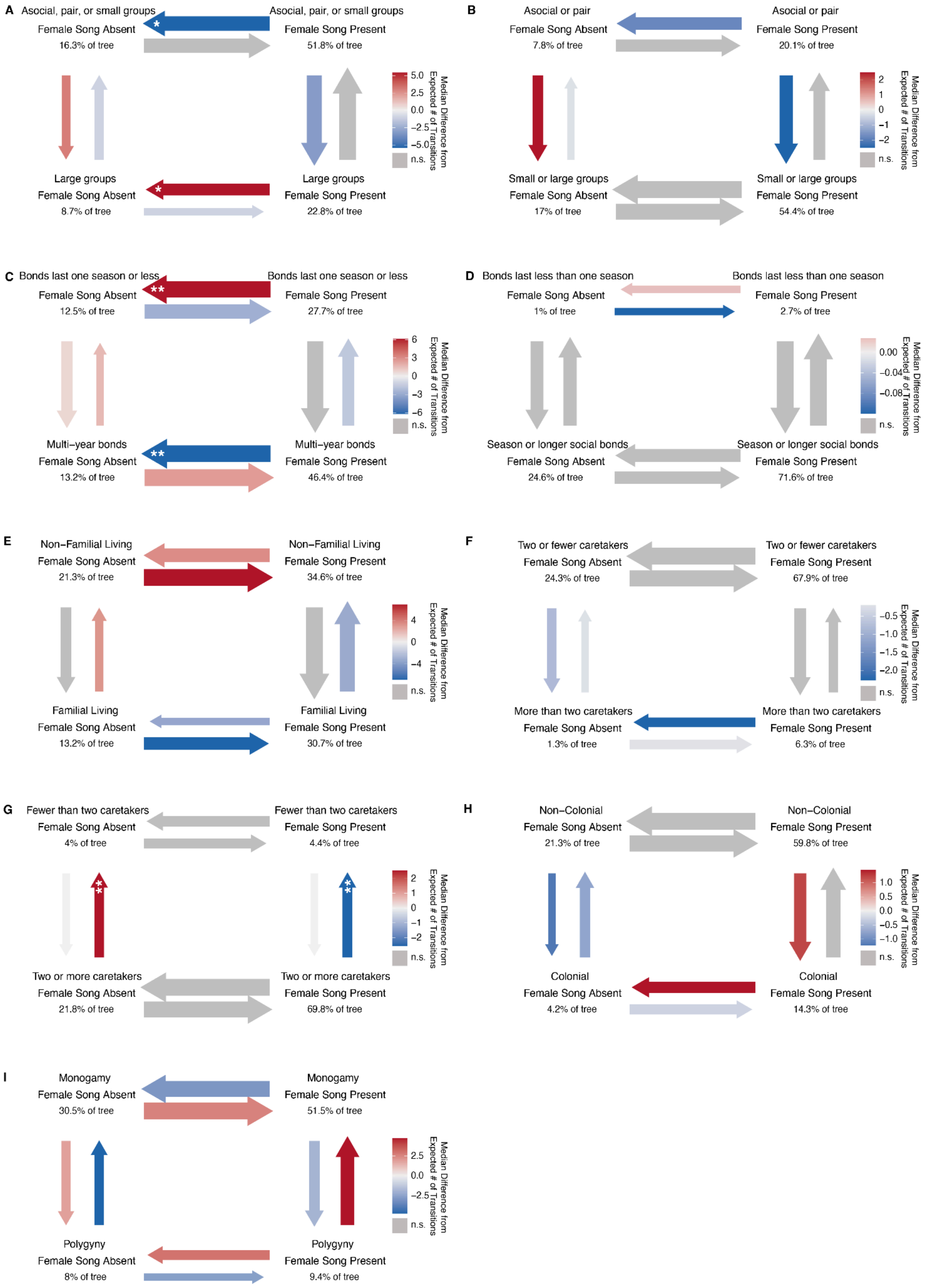

Supplementary Methods Text

For the 523 species classified as Territory level 2 for which we had both female song and cooperative breeding data, we reviewed species accounts in Birds of the World[62] supplemented by Google Scholar literature searches when necessary. To ensure reproducibility, 30 species were independently classified by a second researcher. Our classification criteria for **strong territoriality** included: clear territorial defense behaviors (e.g., "vigorously defending," "chasing intruders," "boundary disputes"), well-defined territory boundaries with minimal overlap, persistent defense throughout breeding season or year-round, active defense mechanisms including displays and vocalizations, and specific territorial use of song. Key indicators included descriptions such as "highly territorial," "aggressively defends," "non-overlapping territories," "responds aggressively to playback," and "maintains territory." **Weak territoriality** was characterized by: limited spatial defense (small areas around nest sites only), tolerance of conspecifics within defended areas, significantly overlapping territories, colonial or semi-colonial breeding, minimal defense behaviors, and mate-focused rather than territory-focused defense. Indicators included "loosely colonial," "overlapping boundaries," "defends only immediate nest site," "soft territoriality," and "tolerant of intruders." This reclassification resulted in 170 species classified as weak territorial (including 114 species with Territory level 1 plus 56 reclassified species with Territory level 2) and 705 species as strong territorial (including 404 species with Territory level 3 plus 301 reclassified species with Territory level 2), with 166 species with Territory level 2 remaining unclassified due to insufficient information.

Supplementary Results Text

Data availability biases

We found no evidence that cooperative breeding species are systematically more likely to have female song presence or absence data ($\chi^2 = 2.18$, $p = 0.14$; Supplementary Table 13). Similarly, coloniality and polygyny showed no significant associations with either female song or cooperative breeding data availability (all $p > 0.05$). However, we detected significant biases related to territoriality, geography, and sexual dichromatism (Supplementary Table 13). Territorial species were significantly more likely to have both female song and cooperative breeding data across all territoriality classifications (all $p < 0.001$). Holarctic species were overrepresented in female song databases ($\chi^2 = 56.8$, $p < 0.001$), and species with greater sexual dichromatism were more likely to have female song classifications ($t = -3.34$, $p < 0.001$). Importantly, among species with female song data, those classified as lacking female song had significantly higher sexual dichromatism ($t = 3.83$, $p < 0.001$), suggesting that dimorphism facilitates confident negative classifications rather than creating false positives for female song presence.

Correlated evolution of female song and other social traits

Several traits, though they did not significantly co-occur with female song in the binary trait co-occurrence analysis (Table ED1), nevertheless appeared to evolve non-independently with female song by affecting its likelihood of being lost or gained (Supplementary Figure 2). We found that loss of female song was more likely to occur than expected in lineages with the largest social group sizes and that female song alongside a smaller group size is a relatively stable combination, with fewer transitions out of the state than expected (Supplementary Figure 2A). Conversely, we found that long social bonds—those lasting longer than one breeding season—tended to promote transition to and maintenance of female song, while shorter social bonds tended to facilitate the loss of female song (Supplementary Figures 2C,D). This is consistent with the trends we observed when analyzing breeding system, considering that longer social bonds also tended to co-occur with cooperative breeding (Supplementary Table 10). Familial living, which is associated with both cooperative breeding and longer social bonds, has a different pattern. We found that familial living tended to reduce the likelihood of either gaining or losing female song, while those likelihoods are increased in lineages with non-familial living (Supplementary Figure 2E). Using the subset of species with data on number of

caretakers from [21] reinforced our finding that lineages that had helpers at the nest, i.e. more than two caretakers, were less likely to lose female song, and those lineages without female song were less likely to increase the typical number of caretakers (Supplementary Figure 2F). Intriguingly, the presence of female song appeared to greatly reduce the likelihood that a lineage would transition to fewer than two caretakers, whereas the loss of biparental care was more likely in the absence of female song (Supplementary Figure 2G). There were not consistent evolutionary transition-rate trends between female song and colonial living or social polygyny (Supplementary Figures 2H,I), although there is an interesting, albeit weak, trend suggesting that social monogamy with female song present as well as social polygyny with female song absent are both attractor states, i.e. that transitions into those states occur more frequently whereas transitions out of those states appear to occur less frequently.

Accounting for other life history predictors

We performed expanded phylogenetic generalized linear model analyses by iteratively adding other life history factors, including geographic region (holarctic vs. tropical; detailed sample sizes provided in Supplementary Table 25), migratory behavior (non-migratory, partial migrant, migratory), familial living (familial vs. non-familial), plumage dichromatism (from sex-specific plumage scores), wing length dimorphism (from sex-specific morphometric data), and latitude (absolute value of the range centroid latitude) to the base models that were best supported for both effects on female song (best model: Female Song ~ Cooperative Breeding * Territoriality + Mass) and effects on cooperative breeding (Cooperative Breeding ~ Female Song * Territoriality). When we performed the stepwise iterative phylogenetic generalized linear model expansion analysis, we found that wing dimorphism and plumage dichromatism improved the model predicting female song presence by >2 AIC, while only familial living substantially improved the model predicting cooperative breeding (Supplementary Tables 21,22). In the expanded models, sexual dimorphism measures (wing: OR = 0.83, plumage: OR = 0.85) were negative predictors of female song presence. Even when including these additional predictors, the cooperative breeding-female song association remained robust (expanded FS model: CB OR = 5.78; expanded CB model: FS OR = 11.74; Supplementary Tables 23,24). In the expanded models with year-round territoriality, geographic factors emerged as significant predictors of female song: tropical species were less likely to have female song presence (OR = 0.67), while species at higher absolute latitudes

were more likely to have female song presence (OR = 1.23). Familial living remained a strong predictor of cooperative breeding in both territorial classifications (OR = 10.62 with year-round territory vs. OR = 14.61 with weak/strong territory).
