# Supplementary material for: Publisher Correction: Territoriality modulates the coevolution of cooperative breeding and female song in songbirds
Source: Nat Ecol Evol. 2026 Mar 18;10(4):822. doi: 10.1038/s41559-026-03046-w (PMC13076191; doi:10.1038/s41559-026-03046-w)
Supplement: Supplementary file 1 — Original, uncorrected Fig. 4 [file 41559_2026_3046_MOESM1_ESM.pdf]

nature ecology & evolution

Supplementary information

Publisher Correction: Territoriality modulates the coevolution of cooperative breeding and female song in songbirds

In the format provided by the authors and unedited

Original, uncorrected figure

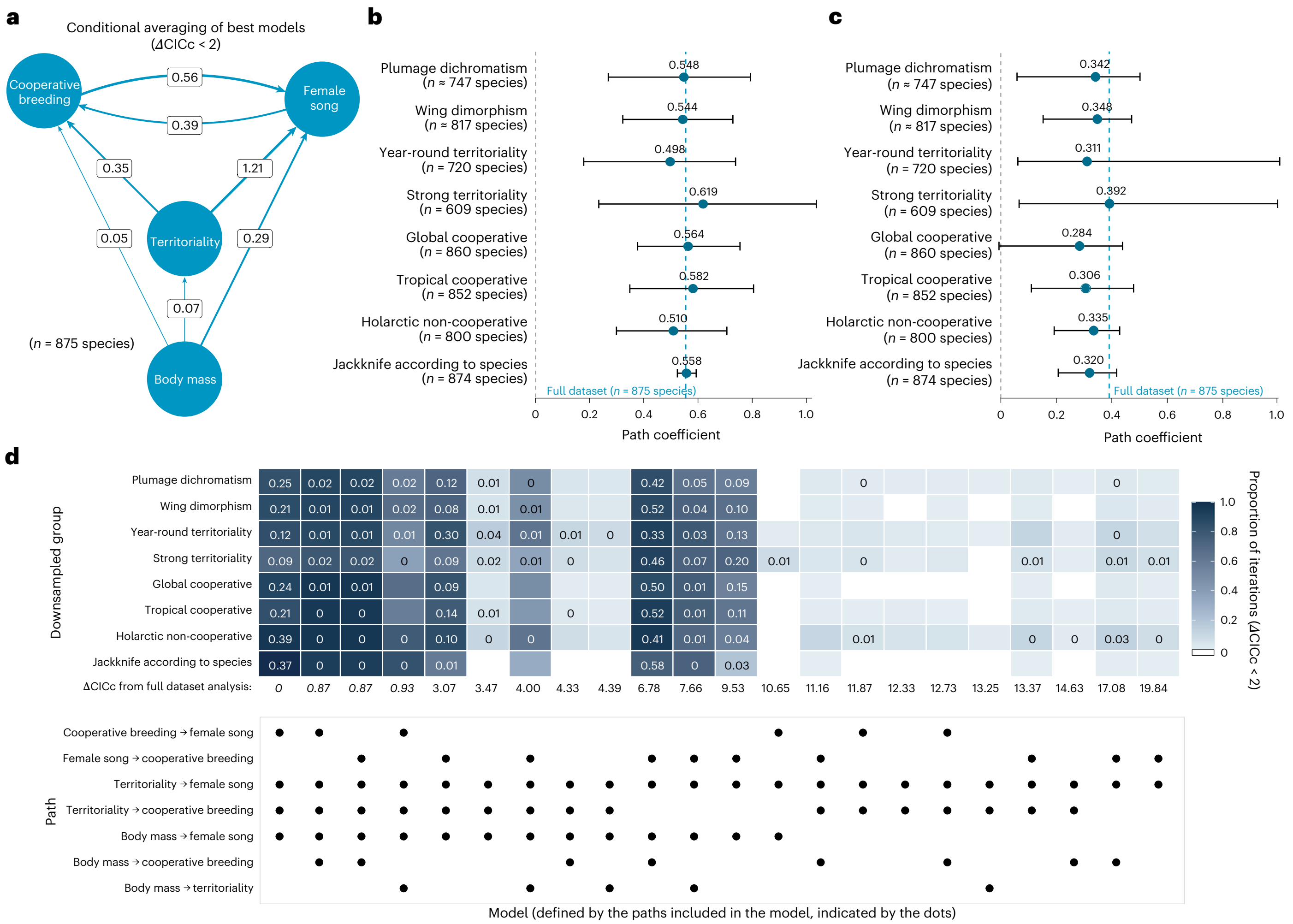

Fig. 4 | The links between cooperative breeding and female song in phylogenetic path analyses are robust to correcting for data availability biases.
